# Utilizing Invasive *Pterygoplichthys pardalis* as a Sustainable Fish Meal Substitute and *Euphorbia hirta* Extract Supplement: Effects on Growth Performance, Organosomatic Indices, Hematological Profiles, and Serum Biochemistry in Chinese Bullfrogs (*Hoplobatrachus chinensis*)

**DOI:** 10.3390/life15010115

**Published:** 2025-01-16

**Authors:** Sontaya Sookying, Phanit Srisuttha, Vipada Rodprasert, Chanthima Chaodon, Wikit Phinrub, Nantaporn Sutthi, Paiboon Panase

**Affiliations:** 1 Division of Pharmacy and Technology, Department of Pharmaceutical Care, School of Pharmaceutical Sciences, University of Phayao, Phayao 56000, Thailand; sontaya.so@up.ac.th; 2 Unit of Excellence Physiology and Sustainable Production of Terrestrial and Aquatic Animals, School of Agriculture and Natural Resources, University of Phayao, Phayao 56000, Thailand; wikit.p@rmutsv.ac.th (W.P.); nantaporn.s@msu.ac.th (N.S.); 3 Department of Applied Thai Traditional Medicine, School of Public Health, University of Phayao, Phayao 56000, Thailand; phanit.sr@up.ac.th; 4 Division of Fisheries, School of Agriculture and Natural Resources, University of Phayao, Phayao 56000, Thailand; vipadanueng001@gmail.com (V.R.); rchantima555@gmail.com (C.C.); 5 Department of Aquaculture and Fishery Products, Faculty of Science and Fisheries Technology, Rajamangala University of Technology Srivijaya, Trang Campus, Trang 92150, Thailand; 6 Department of Agricultural Technology, Faculty of Technology, Mahasarakham University, Mahasarakham 44150, Thailand

**Keywords:** Amazon sailfin catfish, fish meal replacement, herbal extract, frog culture, physiological responses

## Abstract

This research examined the efficacy of substituting commercial fish meal (CFM) with *Pterygoplichthys pardalis* meal (PPM) in *Hoplobatrachus chinensis* diets, with and without *Euphorbia hirta* extract (EHE) supplementation. The study utilized six dietary treatments: a control diet (0% PPM, no EHE) and five experimental diets with varying PPM levels (0%+, 25%+, 50%+, 75%+, and 100%+), each fortified with 300 mg/kg EHE. The experiment spanned 90 days. The analysis revealed that PPM exhibited superior amino acid profiles compared to CFM, both in quality and quantity, while CFM demonstrated higher fatty acid content. The growth metrics showed a significant decline only in the group receiving 100% PPM replacement with EHE supplementation. Most organosomatic indices remained consistent across the treatments, with the exception of intraperitoneal fat, which decreased in all EHE-supplemented groups. Blood parameters, including white blood cells, red blood cells, and hematocrit, along with serum proteins (total protein, globulin, and albumin), displayed an upward trend in all EHE-supplemented groups. The 50%+ and 75%+ PPM replacement groups exhibited significantly elevated serum glucose levels (*p* < 0.05). Liver enzymes (alanine transaminase and aspartate transaminase) showed no significant variations among the treatments. The results indicate that PPM can serve as an effective replacement for up to 75% of CFM in *H. chinensis* feed, without compromising their growth performance. Moreover, supplementing with EHE helps to enhance essential biochemical indices in the body, without adversely affecting liver function. This investigation offers valuable perspectives on the development of sustainable aquaculture feed and the potential application of invasive fish species in aquatic animal nutrition.

## 1. Introduction

The suckermouth armored catfish, belonging to the genus *Pterygoplichthys* and the family Loricariidae, has become a highly invasive non-indigenous species in Asia [1,2] and Southeast Asia [3], particularly in Thailand [4]. Originally introduced into the region for ornamental purposes and as a way to clean aquaria, *Pterygoplichthys pardalis* was subsequently released into natural waterbodies such as rivers and streams, where it bred expeditiously [5,6]. The factors contributing to the rapid propagation of this fish species include its high tolerance to poor water quality, low susceptibility to diseases, rapid reproduction rate, and, importantly, its lack of popularity as a food source among the local population. The rapid proliferation of *P. pardalis* has had detrimental consequences for native fish species due to several impacts. Firstly, they compete intensely for food and habitat resources due to their ability to consume diverse organic materials, often resulting in reduced food availability for indigenous species. Secondly, their burrowing activities lead to habitat degradation, causing erosion and destabilizing environments that are crucial for native species’ reproduction and shelter. Thirdly, their aggressive behavior and prolific reproduction facilitate the displacement of native species, thereby diminishing the biodiversity and altering aquatic community structures. Lastly, they pose a health threat by potentially introducing parasites and diseases that the native fauna are ill equipped to resist. This alien species also destroys spawning areas, especially those where demersal eggs are deposited [7]. According to a report by Thalathiah and Palanisamy, the native Cyprinidae have also been negatively affected by the presence of these invasive catfish [8]. This predation behavior not only impacts native fish populations but also positions *P. pardalis* as a potential vector for non-native parasites [9,10]. Moreover, the report by Lai et al. revealed that *Pterygoplichthys* spp. can be found in brackish water systems. This may be one reason that this species can reproduce rapidly [11].

Fish meal is a well-known ingredient used in aquaculture feed to provide essential nutrients to farmed fish and shrimp. Many species of farmed fish, including Nile tilapia and catfish, as well as frogs and other domestic animals, rely on fish meal for their protein requirements [12,13,14]. The availability and price of fish meal depend on many factors, such as the abundance of fish, environmental regulations, and the global demand for aquaculture and animal feed. Sustainability concerns stemming from issues like overfishing and the need for environmentally responsible practices have prompted a significant shift in the aquaculture and animal feed industries. This transformation is characterized by a growing emphasis on utilizing alternative protein sources that are both eco-friendly and economically viable, such as plant-based proteins [15] and insects [16], to reduce the reliance on fish meal. Indeed, recognizing that *P. pardalis* is not generally consumed directly by humans, some studies have explored alternative approaches, including producing post-harvest and value-added products like fish biscuits. One approach to effectively reduce the *P. pardalis* populations in natural waters and fishponds is converting *P. pardalis* into fish meal for commercial fish farming, which could be a win–win solution, benefiting both the control of invasive species and the aquaculture industry while also promoting environmental sustainability [13,17].

The Chinese bullfrog (*Hoplobatrachus chinensis* Osbeck, 1765) was previously classified as *Hoplobatrachus rugulosus*, a change that was implemented for specific taxonomic reasons. The updated classification of *chinensis* was reflected in the “Amphibian Species of the World 6.2”, an authoritative online reference [18]. This species plays a significant role in the overall biodiversity of East and Southeast Asia, including regions such as China, Taiwan, Vietnam, Thailand, Laos, and other neighboring areas. In 2022, Thailand recorded the substantial production of this frog species, totaling 1810 tons, with significant economic value of THB 125,313,000 (USD 3.9 million), according to data from the Fisheries Development Policy and Planning Division [19]. Currently, there is a growing trend among farmers to raise Chinese bullfrogs in higher densities, which has led to increased stress among the frogs, resulting in a slower growth rate and instances of cannibalism due to insufficient food resources [20]. Farmers sometimes resort to antibiotics to treat various diseases in frogs, but this practice can give rise to unintended consequences, including the accumulation of chemical additives in the frogs. Additionally, many pathogens may develop resistance to antibiotics over time, further complicating the management of this frog species’ health [21].

Nowadays, the use of herbal extracts as dietary supplements in aquatic animal farming or culture is a topic of interest in scientific research and aquaculture practices for several reasons, such as their antibacterial and antifungal properties, immunostimulatory effects, stress reduction, improved growth performance, reduced reliance on chemicals, and consumer preferences, as well as research and innovation [22,23,24,25]. Thailand has a variety of herbs that can be used in aquatic animals. One of them is garden spurge (*Euphorbia hirta* L.). This plant is a member of the Euphorbiaceae family, with its natural habitat in Southeast Asia. Garden spurge is gaining attention as a promising candidate for pharmaceutical applications and is extensively employed as a traditional herbal remedy in tropical regions. The plant has yielded various bioactive compounds, including flavonoids, terpenoids, phenols, essential oils, and numerous constituents that have been successfully extracted and identified [26,27]. The positive effects of this plant have been reported; the incorporation of EHE into the diets of common carp (*Cyprinus carpio* Linnaeus, 1758) infected with *Aeromonas hydrophila* resulted in significant improvements in their red blood cell counts (RBC), white blood cell counts (WBC), and hemoglobin levels, as well as increased antibody levels, enhanced lysozyme activity, and improved phagocytic activity [28]. In addition, Panase et al. demonstrated that the administration of 300 mg/kg of *E. hirta* leaf extract had a positive impact on the growth performance of hybrid catfish (*Clarias macrocephalus* × *C. gariepinus*), i.e., the average daily gain (ADG), specific growth rate (SGR), feed efficiency (FE), feed conversion ratio (FCR), protein efficiency ratio (PER), and survival rate (SR), as well as organosomatic indices, i.e., the hepatosomatic index (HSI), intraperitoneal fat (IF), and viscerosomatic index. The dose did not affect the hematological parameters compared to the control and higher-dose groups [27].

In light of the information outlined above, including the economic significance of *H. chinensis* farming, the challenges associated with costly fish meal, the distribution of *P. pardalis* in natural water sources, and the potential benefits of EHE, this study was conducted to investigate the impact of fish meal replacement with PPM and supplementation with 300 mg/kg EHE on the growth performance, serum biochemistry, and hematological indices of *H. chinensis*, as no previous research or report has been undertaken on this subject.

## 2. Materials and Methods

### 2.1. Ethical Statement

The scientific procedures were conducted according to the guidelines for the animal care and use of the University of Phayao and approved (20 December 2021) by the Committee of Institutional Animal Care, University of Phayao, Thailand (ID: 640104033).

### 2.2. Preparation of EHE

The dried aerial parts of *E. hirta* were purchased from a trusted herbal store with GMP quality in Bangkok. In this study, ethanol was used as the extraction solvent due to its relatively safe and environmentally friendly properties as an organic solvent. Additionally, it is easy to evaporate to reduce the volume of the extract. The plant was ground into a powder, after which the crude powder was macerated using 95% commercial-grade ethanol at the ratio of 1:12 *w*/*v* of plant material and solvent, respectively. The sample was stirred at 100 rpm for 72 h. The mixture was then filtered to collect the ethanol part. The plant residue was then macerated once more using a 1:4 ratio of plant to ethanol. The same extraction technique was performed for three consecutive days. After filtration, the filtrates were gathered, and the solvent was removed under low pressure at 40–50 °C using a rotary evaporator. The resulting extract was carefully stored in a tight container, protected from light, and kept in a refrigerator.

### 2.3. Phytochemical Screening of EHE

In this investigation, standardized procedures were followed, in line with Pant et al., Hartanti and Cahyani, and Shaikh and Patil [29,30,31]. Various screening tests were conducted for the presence of phytochemicals in EHE. Alkaloids were detected using Dragendorff and Mayer’s reagent, while the Liebermann–Burchard test was employed to evaluate the potential existence of steroids and terpenoids. For the identification of carbohydrates, deoxy sugars, and α, β unsaturated 5-membered lactone rings, the Molisch, Keller–Killiani, and Kedde tests were utilized, respectively. To screen for cyanogenic glycosides, the sodium picrate technique was employed, and saponin screening was conducted using a frothing test. The presence of phenolic compounds was investigated using the ferric chloride technique, while the detection of flavonoids was accomplished through Shinoda’s test. Coumarins were observed using the sodium hydroxide paper test. Finally, anthraquinones were identified utilizing a modified Borntrager approach.

### 2.4. Determination of Total Phenolic Content in EHE

The content of total phenolics in the extract was determined using the Folin–Ciocalteu colorimetric method, following the method outlined by Chandra et al., with slight modifications [29]. Gallic acid was used as a standard phenolic compound. The standard calibration curve of gallic acid was constructed in the range of 3.125–200 μg/mL. Stock and working solutions of gallic acid and EHE were produced using 80% methanol. For analysis, 20 μL of gallic acid or EHE was pipetted into a 96-well plate, followed by 50 μL of 10% Folin–Ciocalteu reagent in water. The plate was shaken and kept in the dark for 5 min. Then, 80 μL of sodium carbonate solution (7.5% *w*/*v* in water) was added. The reaction was kept in the dark for 30 min and the absorbance was measured at 760 nm. The standard calibration curve was plotted between the concentrations and absorbance (y = 0.0044x + 0.0041, *R*^2^ = 0.9958). The total phenolic content was determined and calculated as milligrams of gallic acid equivalents per gram of EHE (mg GAE/g extract). All determinations were carried out in triplicate.

### 2.5. Determination of Total Flavonoid Content in EHE

The aluminum chloride colorimetric method was modified from the method of Chandra et al. for the determination of the total flavonoid content [32]. Quercetin was used as a standard flavonoid compound. The quercetin standard and EHE were prepared by dilution with 80% methanol. The standard calibration curve of quercetin was a plot of the absorbance against the concentration (3.125–200 μg/mL). For analysis, 20 μL of the quercetin solution or test sample was pipetted into a 96-well plate. Then, 50 μL of 2% aluminum chloride in water was added. The mixture was kept in the dark for 5 min. Thereafter, 50 μL of 50 mM sodium hydroxide in water was added. The reaction was kept in the dark for a further 30 min, and the absorbance at 415 nm was measured. The flavonoid content was calculated as quercetin equivalents per gram of extract (mg QE/g extract) on the basis of the standard calibration curve of quercetin (y = 0.0037x + 0.0011, *R*^2^ = 0.9999). All determinations were carried out in triplicate.

### 2.6. Determination of Antioxidant Capacity

The capacity of EHE to directly react with free radicals was determined using the 2,2-diphenyl-1-picrylhydrazyl (DPPH) assay, which was slightly modified from Bello et al. [33]. Stock solutions of ascorbic acid (2000 μg/mL) and DPPH (0.1 mM) were prepared using methanol. Working solutions of ascorbic acid and EHE were produced using 80% methanol. The assay was performed in a 96-well plate. Next, 100 μL of ascorbic acid or EHE was pipetted into each well, followed by 100 μL of DPPH solution. The plate was meticulously shaken and kept in a dark place for 30 min. Then, the absorbance (A) at 517 nm was measured. The % radical scavenging was calculated using the formula % radical scavenging [(Abs_blank_ − Abs_sample_)/Abs_blank_] × 100 and plotted against the concentration. The antioxidant capacity was reported as the IC_50_. All determinations were carried out in triplicate.

### 2.7. Preparation of Fish Meal from Pterygoplichthys pardalis

Following the methods outlined by Panase et al. [13], fish were sourced from private fish farms in Phayao Province, Thailand, by local farmers. In total, more than 400 g of *P. pardalis* were collected, and they underwent a thorough washing process after removing their visceral organs. Subsequently, the fish were roasted and left to air dry completely under sunlight for a period of five days. To eliminate any remaining moisture, all specimens were further subjected to storage at 70 °C for 24 h. After this step, the specimens were finely ground into a powder using a meat grinder, sifted through a blue nylon net with a mesh size of 425 μm, and then stored in a sterile environment. The final yield (dry weight) was 330 g per kilogram of wet weight.

### 2.8. Preparation of EHE-Supplemented PPM

The experimental diets, containing 0%, 25%, 50%, 75%, and 100% PPM, were sprayed with EHE diluted in deionized water at a dose of 300 mg/kg of diet in a drum mixer and left to dry. The sprayed pellets were then coated with a 4% agar solution at a concentration of 10 mL/kg and air-dried again. They were stored in sterile containers at room temperature for seven days. New batches were prepared weekly [22].

### 2.9. Biochemical Composition Analysis of PPM and CFM

The fish meal obtained from the production process involving *P. pardalis*, as described above, was analyzed together with a commercial fish meal acquired from an agricultural supply store in Chiang Mai, Thailand. A proximate analysis was conducted, encompassing assessments of the moisture, ash, crude protein, and crude fat, employing the standardized methods outlined by the AOAC [34]. Specifically, the Kjeldahl distillation method was applied to determine the crude protein content, while the crude lipid content was ascertained through Soxhlet extraction, and the ash content was determined using drying methods. The findings from these analyses are presented in Table 1.

The biochemical compositions of the PPM and CFM were analyzed. The amino acid profiles were also assessed using an amino acid analyzer from the Bio 30^+^ series by Biochrom Ltd., Cambridge, UK, following an in-house method, TE-CH-372, inspired by the Official Journal of the European Communities [35]. Meanwhile, the analysis of the fatty acid profiles was performed via gas chromatography (GC) using an in-house method, TE-CH-208, which is derived from the AOAC 996.06 standards [36]. Both analyses were carried out by the Analytical Service at the Central Laboratory (Chiangmai, Thailand) Co., Ltd.

### 2.10. Experimental Frogs, Conditions for Acclimatization, and Experimental Design

Seven days before the experiment, 55-day-old juvenile frogs were obtained from the Faculty of Agriculture and Natural Resources, University of Phayao, Thailand. They were then raised in cages to allow the juvenile frogs to adapt to the environment. The juvenile frogs were acclimatized in a net cage that was 200 cm × 300 cm × 50 cm in size with an oxygen generator for 2 weeks under laboratory conditions. The water was changed at 3 intervals at a rate of 50% of its total volume. Water quality parameters were monitored at 7:00 a.m. daily throughout the acclimatization period. The temperature was maintained at 28.6 ± 2.12 °C, with a dissolved oxygen (DO) concentration of 7.2 ± 0.16 mg/L and a pH of 7.2 ± 0.35. Measurements were conducted using multi-probes from the HORIBA U50 series, ensuring that the conditions were similar to those described in the study by Thip-uten et al. [37].

The frogs were fed with a commercial diet twice a day at 9:00 a.m. and 5:00 p.m. For the experiment in this study, a completely randomized design was used. Initially, the juvenile frogs were weighed before the experiment, and the average initial weight was 7.06 ± 12.32 g; after this, they were divided into 6 triplicate groups and placed into net cages that were 100 cm × 200 cm × 50 cm in size, with 20 juvenile frogs per cage. Each group was fed with their own prepared diet (Table 2) twice daily (9:00 a.m. and 5:00 p.m.) at 7% of their body weight per day for 90 days [38].

### 2.11. Growth Performance and Survival

At 15-day intervals, the frogs in each cage were weighed to regulate the feed quantities and assess their growth performance. The growth parameters included weight gain (WG), ADG, SGR, FCR, PER, and SR. The calculations for these parameters were performed using the provided equations [39]:

WG (g) = final weight (g) − initial weight

ADG (g/day) = [{final weight (g) − initial weight (g)}]/experimental days

SGR (%/day) = [{ln final weight (g) − ln initial weight (g)}/experimental days] × 100

FCR = total feed fed (g)/weight gain (g)

PER = wet weight gain (g)/crude protein fed

SR (%) = [number of surviving frogs/initial number of frogs] × 100

### 2.12. Organosomatic Indices

At the end of the experiment, three frogs from each cage (replicate) were randomly chosen and subjected to anesthesia using isoflurane, following the protocol outlined by Smith and Stump [40]. The visceral organs, including the liver, spleen, kidney, fat, and intestine, were carefully separated for further analysis to calculate the organosomatic indices. These indices encompassed the hepatosomatic index (HSI), spleenosomatic index (SSI), renosomatic index (RSI), intestinosomatic index (ISI), and intraperitoneal fat (IF). The calculations were performed as follows [41]:

HSI (%) = [liver weight (g)/body weight (g)] × 100

SSI (%) = [spleen weight (g)/body weight (g)] × 100

RSI (%) = [kidney weight (g)/body weight (g)] × 100

ISI (%) = [intestine weight (g)/body weight (g)] × 100

IF (%) = [intraperitoneal fat weight (g)/body weight (g)] × 100

### 2.13. Study of Hematological Indices

This study randomly selected nine frogs from each group (3 frogs per replication). Before the blood collection process, each frog was subjected to anesthesia using isoflurane, following the method detailed by Smith and Stump [40]. On day 90 of the experimental period, blood samples were obtained from each frog through cardiac puncture, following the technique outlined by Heatley et al. [42]. Blood samples were drawn from the cardiac puncture (0.4 mL) using syringes equipped with 26G needles, and these samples were collected in K3 EDTA tubes. To determine the red blood cell count, a blood sample was diluted to 1:300 in a 0.85% NaCl solution, while the hematocrit (Hct) was determined using the microcentrifuge method. Furthermore, a blood sample was diluted to 1:50 in a 2% acetic acid solution to assess the white blood cell count. The determination of both the RBC and WBC was conducted manually using a Neubauer chamber, as described by Rehulka [43].

### 2.14. Serum Biochemistry Study

On day 90 of the feeding trial, the serum biochemistry was examined. After assessing the growth indices and survival rates, 9 frogs from each group, with 3 frogs per replication, were randomly selected for blood collection. Blood samples (0.8 mL per frog) were collected via cardiac puncture using non-heparinized syringes. These blood samples were transferred to micro-centrifuge tubes and allowed to clot at room temperature for a period of 4 h before they were utilized for serum collection. Subsequently, they were subjected to centrifugation at 5000 rpm for 15 min at 25 °C. The resulting supernatants were carefully transferred to sterile serum tubes and stored at −20 °C until use, with a maximum storage duration of 7 days. The frozen serum samples were later transported to the Chiang Mai Veterinary Laboratory Centre Limited Partnership in Chiang Mai, Thailand, for analyses. Serum indices, including alanine transaminase (ALT), aspartate transaminase (AST), glucose, total protein, albumin, and globulin, were assessed using an analytical chemistry analyzer (PC400, HORIBA, Kyoto, Japan).

### 2.15. Statistical Analysis

Before conducting the analyses of the growth parameters and hematological and serum biochemistry indices, the data were normalized and assessed for homogeneity of variance. Subsequently, all parameters were subjected to a one-way analysis of variance (ANOVA), followed by Tukey’s post-hoc test at a significance level of 95% (*p* < 0.05). The results are presented as mean values along with their corresponding standard deviations. Statistical analyses were carried out using STATA version 18.

## 3. Results

### 3.1. Phytochemical Composition, Total Phenolic Content, Total Flavonoid Content, and Antioxidant Capacity of EHE

The phytochemical constituents of EHE observed in the screening tests were terpenoids, coumarins, phenolics, and carbohydrates (Table 3). The total phenolic content, total flavonoid content, and IC_50_ of EHE were 573.58 ± 29.83 mg GAE/g extract, 39.17 ± 2.90 mg QE/g extract, and 11.06 ± 6.54 μg/mL, respectively (Table 4).

### 3.2. Amino Acid and Fatty Acid Profiles of PPM Meal and CFM

The analysis of the amino acid profile revealed that the PPM contained significantly higher levels of each essential amino acid compared to the CFM, with amounts that were nearly twice as high. Similarly, the non-essential amino acids were also present in higher quantities in the PPM than in the CFM. When considering the combined results of both essential and non-essential amino acids, it was observed that the PPM contained 1.67 times more amino acids than the CFM (Table 5).

In contrast to the amino acid profile, the fatty acid profile showed differing results, both in terms of types and quantities. The analysis indicated that the CFM contained a greater variety and quantity of fatty acids than the PPM. Specifically, the levels of saturated fatty acids, unsaturated fatty acids, monounsaturated fatty acids, and polyunsaturated fatty acids in the CFM were 4.85, 3.54, 4.11, and 4.93 times higher, respectively. Additionally, the amounts of omegas 3, 6, and 9 in the CFM exceeded those in the PPM by 13.21, 1.74, and 2.37 times, respectively (Table 6).

### 3.3. Growth Performance

**Figure 1:**
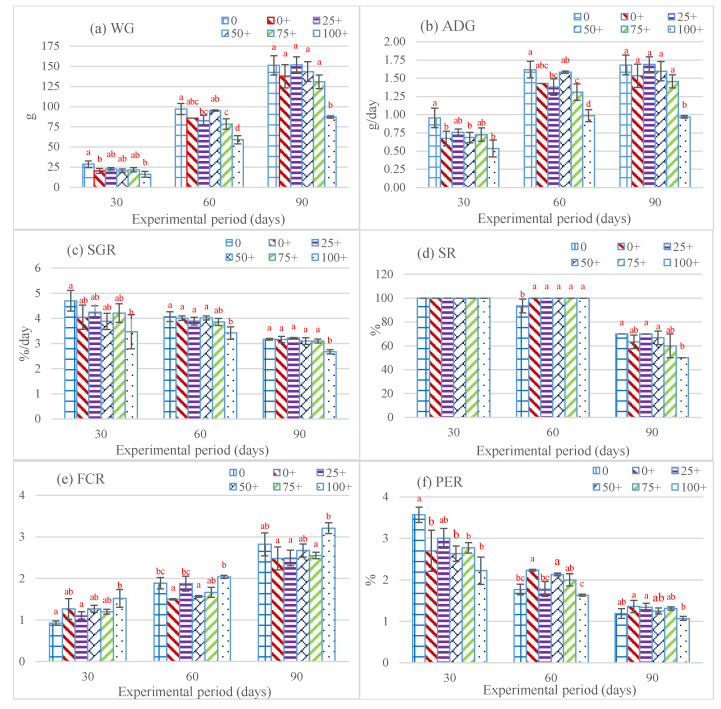
Growth performance of *Hoplobatrachus chinensis* fed with fish meal replacement at six different levels of *Pterygoplichthys pardalis* meal and supplemented with *Euphorbia hirta* leaf extract for 90 days; WG = weight gain (**a**), ADG = average daily growth (**b**), SGR = specific growth rate (**c**), SR = survival rate (**d**), FCR = feed conversion ratio (**e**), and PER = protein efficiency ratio (**f**). Different letters indicate significant differences (*p <* 0.05).

After the 90-day feeding period, the findings related to the growth performance of *H. chinensis*, which were exposed to six distinct experimental diets, were as follows. Notably, significant differences (*p* < 0.05) in WG emerged as early as day 30 of the experimental period. The lowest WGs were observed in 100+ at days 60 and 90, while the 75+ group showed a significant difference from the other groups only at day 60. At the end of the study, there were no differences between the 0, 0+, 25+, 50+, and 75+ groups (Figure 1a). Likewise, ADG displayed a parallel trend to that of WG, showing significant differences starting from day 30 and persisting through days 60 and 90 of the experiment. Notably, the highest ADG values were consistently observed in the 0 group, while the 100+ group consistently exhibited the lowest ADG values throughout the study period (Figure 1b). Like the SGR and PER, the 100+ group exhibited the lowest values on days 30, 60, and 90 of the experiment (Figure 1c,f). The 0 group showed the highest SGR throughout the study period, as it revealed the highest PER only at day 30, which then decreased to lower levels compared to the other groups at days 60 and 90. The FCR exhibited the highest levels in the 100+ group throughout the experimental period, and it differed significantly from the 0+ group at days 60 and 90 (Figure 1e). At day 60, the SR in the 0 group was significantly lower than those in the groups that received EHE (Figure 1d). The lowest SR, however, was observed in the 100+ group at the end of the study.

### 3.4. Organosomatic, Hematological, and Serum Biochemical Indices

**Figure 2:**
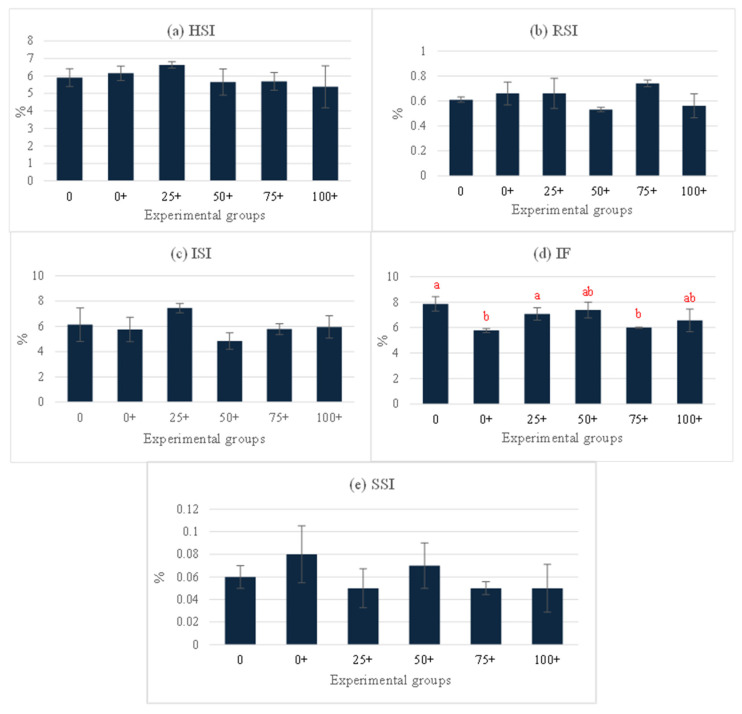
Organosomatic indices of *Hoplobatrachus chinensis* fed with fish meal replacement at six different levels of *Pterygoplichthys pardalis* meal and supplemented with *Euphorbia hirta* leaf extract for 90 days; HSI = hepatosomatic index (**a**), RSI = renosomatic index (**b**), ISI = intestinosomatic index (**c**), IF = intraperitoneal fat, (**d**) and SSI = spleenosomatic index (**e**). Different letters indicate significant differences (*p <* 0.05).

**Figure 3:**
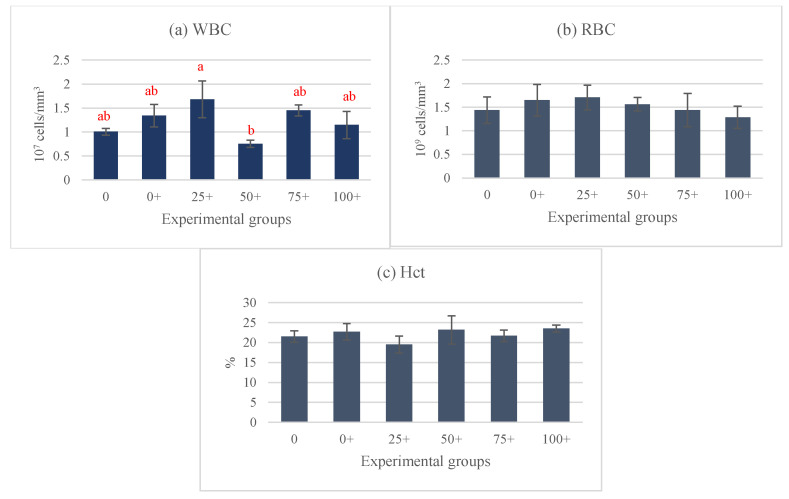
Hematological indices of *Hoplobatrachus chinensis* fed with fish meal replacement at six different levels of *Pterygoplichthys pardalis* meal and supplemented with *Euphorbia hirta* leaf extract for 90 days; WBC = white blood cell count (**a**), RBC = red blood cell count, (**b**) and Hct = hematocrit (**c**). Different letters indicate significant differences (*p <* 0.05).

**Figure 4:**
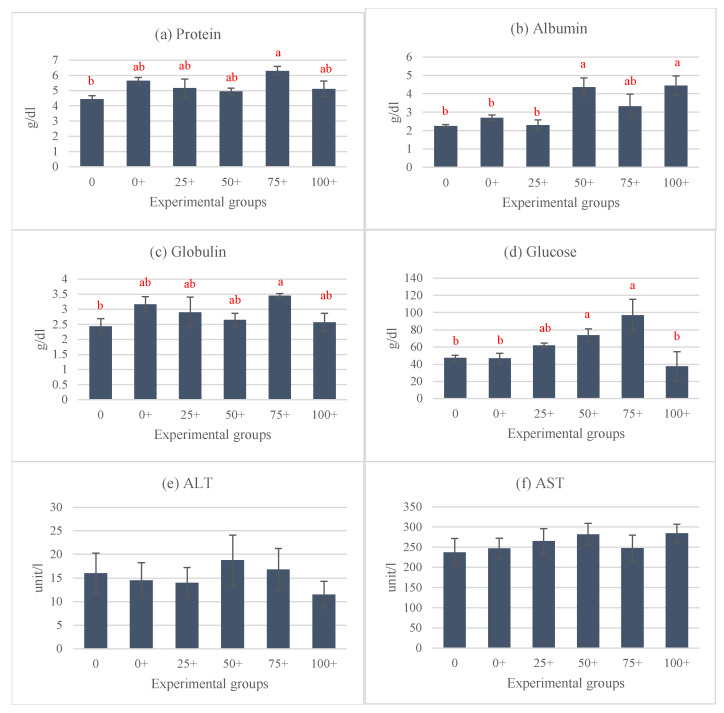
Serum biochemical indices of *Hoplobatrachus chinensis* fed with fish meal replacement at six different levels of *Pterygoplichthys pardalis* meal and supplemented with *Euphorbia hirta* leaf extract for 90 days: total protein (**a**), albumin (**b**), globulin (**c**), glucose (**d**), ALT—alanine transaminase (**e**), and AST—aspartate transaminase (**f**). Different letters indicate significant differences (*p <* 0.05).

At the end of the experiment, the organosomatic indices of *H. chinensis* showed no significant differences, except in the case of IF (Figure 2a–e). Notably, the highest IF level was observed in the 0 group, while the lowest levels were determined in the 0+ and 75+ groups (Figure 2d). Hematological indices, including the WBC, RBC, and Hct were assessed in this study (Figure 3a–c). Among these parameters, only the WBC exhibited significant differences, while the rest of the parameters did not show any significant differences between the groups. The highest WBC level was observed in the 25+ group, whereas the lowest level was noticed in the 50+ group. Interestingly, the WBCs in the remaining four groups (0, 0+, 75+, and 100+) did not exhibit significant differences from each other (Figure 3a). This study also examined various serum biochemical indices, including the total protein, albumin, globulin, glucose, ALT, and AST. The findings indicated that the total protein, albumin, globulin, and glucose were influenced by the experimental diets. Interestingly, the 75+ group exhibited a significantly higher total protein level compared to the 0 group, and there were no significant differences between the other five groups that received EHE (Figure 4a). Additionally, regarding serum albumin, low values were observed in the 0, 0+, and 25+ groups, while higher levels were discovered in the higher-dose groups (Figure 4b). At 75% replacement, the serum albumin did not significantly differ from that of the 0 group. For the serum globulin, the 0 group exhibited the lowest level in comparison to the other five groups. The highest globulin level was observed in the 75+ group (*p* < 0.05 vs. 0 group) (Figure 4c). The serum glucose level was not affected by EHE, but replacement with 75% of PPM increased the level of serum glucose in a dose-dependent manner. The highest dose of PPM exhibited no effect on the serum glucose level (Figure 4d). Neither ALT nor AST showed significant differences among the experimental groups (Figure 4e,f).

## 4. Discussion

In this study, the focus was on the Chinese bullfrog, *H. chinensis*, due to its important role in aquaculture. The first consideration is that it is an economically valuable species on a global scale, and the second is its adaptability to diverse environmental conditions. Additionally, this frog species is well suited for high-density farming operations. It exhibits a rapid growth rate and is a good source of high-quality meat for consumption. However, despite these advantages, farmers face high production costs, particularly regarding feed expenses. Thus, feed cost reduction is an important aspect for problem-solving. Fish meal replacement with local alternatives to lower costs and increase feed production has gained the interest of aquafeed researchers. Nevertheless, farmers, in the context of rising production costs, are still encountering significant challenges, particularly with regard to feed expenses. Consequently, the pursuit of cost-effective solutions that reduce feed costs has emerged as a paramount strategy to address this issue. In this regard, aquafeed researchers are placing a considerable emphasis on the substitution of fish meal with locally available and cost-effective alternatives, aiming to achieve more economical and sustainable feed production practices.

For instance, the use of commercial feed (30% protein) in combination with black soldier fly larvae, both in dried form (54.5% protein) and fresh form (16.2% protein), has been explored for the feeding of Thai frogs (*Rana rugosa*). The results indicated that a combination of 50% fresh black soldier fly larvae and 50% commercial feed could be successfully applied for Thai frog production on a farm scale in Vietnam [44]. In their study, Klahan et al. investigated a feeding regime for Thai frogs, administering frog feed in the first month, alternating with catfish feed in the second month, and returning to frog feed in the third month. This approach revealed a suitable feeding regime [45].

According to the research findings obtained in this study, replacing 75% of CFM with meal from an alien fish species (*P. pardalis*) and mixing it with 300 mg/kg of EHE did not result in any significant changes in growth indices when compared to the other groups. It is also worth noting that the group fed with 100% PPM as a fish meal replacement exhibited the lowest growth indices among all experimental groups. The selection of the 300 mg/kg concentration of EHE in this research was based on the results given in a previous publication by the authors. In a previous study by Panase et al., it was observed that this specific concentration of EHE was the most effective in improving growth performance, enhancing the hematological and organosomatic indices in hybrid catfish [27]. Using 75% replacement by PPM did not show any adverse effects when compared to the control group. This research’s findings align with previous reports that have demonstrated the successful utilization of diets incorporating meat meal with replacement levels ranging from 30% to 70% as a substitute for other types of fish meal. This acceptance of meat meal diets has been observed in both omnivorous and carnivorous fish species [46]. Based on the analyses of the amino acid and fatty acid profiles, it was observed that the qualitative and quantitative parameters of the essential amino acid profile of PPM were approximately twice those of the CFM. The fatty acid profile of PPM was notably inferior to that of the CFM, with deficiencies observed in MUFA, PUFA, and omega 3, which were lower by factors of 3.15, 4.93, and 13.21, respectively. These findings suggest that CFM holds greater significance, likely due to the fish meal being sourced from one or a limited number of species. These findings are consistent with the research conducted by Panase et al., which demonstrated that replacing fish meal with PPM in the diet of juvenile Mekong giant catfish (*Pangasianodon gigas* Chevey, 1931) at up to 75% showed no adverse effects, while increasing the replacement percentage beyond this threshold resulted in a declining trend in terms of growth performance [13]. In addition, in the juvenile guppy (*Poecilia reticulata*), the growth indices were not significantly different after being fed 100% fish meal replacement with Sailfin catfish meal from genus *Pterygoplichthys* [17]. Moreover, in the 100+ group, the CFM was entirely replaced with PPM, where PPM refers to fishmeal produced from a single species of freshwater fish, while the CFM is derived from various marine fish species. When examining the fatty acid profiles, it was evident that the CFM was richer in both the types and quantities of fatty acids. Notably, it contained significantly higher levels of unsaturated fatty acids, such as omega 3, which were more than 13 times higher than those in the PPM. Ibrahim et al. reported that omega 3 has stress-reducing effects and can enhance the immune response in Nile tilapia; however, there are no studies concerning these effects in frogs [47].

The current comprehensive study aimed to substantiate the benefits of incorporating PPM as a practical substitute for CFM, including not only the assessment of the growth performance but also the examination of the organosomatic, hematological, and serum biochemical indices. This holistic approach was adopted because these indices can provide valuable insights into changes in aspects such as the visceral organ sizes, and they are influenced by many factors, including the water quality, dietary composition, stocking density, and stress, among others [48,49,50]. Regarding the organosomatic indices, the results obtained herein demonstrated no significant differences between the groups for any parameter except IF. Across all groups that were fed with 300 mg/kg of EHE, a consistent trend of reduced IF was observed in comparison to the group that did not receive the extract (0 group). This trend suggests that EHE may influence lipid metabolism, which contributes to promoting growth. Furthermore, this might be affected by the antioxidant capacity of EHE. This is consistent with previous research findings, which indicated that the administration of an antioxidant, astaxanthin, could help to reduce the IF in juvenile largemouth bass *(Micropterus salmoides*) [51]. Notably, a study by Li et al. revealed that Siberian ginseng (*Acanthopanax senticosus*) extract had the capacity to reduce the free fatty acids, triglycerides, total cholesterol, and liver LDL-C, while simultaneously increasing HDL-C, in the liver of Nile tilapia (*Oreochromis niloticus*) [52]. Therefore, it is plausible that the inclusion of 300 mg/kg of EHE may lead to a reduction in lipid synthesis, an enhancement in lipid catabolism through the regulation of lipid transport, and a decrease in fat deposition within the body. Moreover, feeding with a lotus (*Nelumbo nucifera*) leaf alcoholic extract was reported to increase the serum HDL-C and reduce the serum LDL-C in grass carp, *Ctenopharyngodon idellus* [53]. In this study, it is noteworthy that most parameters related to the organosomatic indices did not exhibit significant differences between the groups. This observation is consistent with the findings of a study conducted by Panase et al., which also demonstrated that all organosomatic indices in juvenile Mekong giant catfish remained stable and were not significantly altered when the fish were fed with diets that ranged from 0% to 100% fish meal replacement with *P. pardalis* meal [13].

In this study, fish meal replacement by PPM at all levels revealed no negative effects on the hematological indices. Conversely, the effect on the WBC was found to be positive, possibly due to certain bioactive components that can stimulate immune function, as previously reported by Pratheepa and Sukumaran [28]. Their study demonstrated that *E. hirta* leaf extract exhibited the effect of increasing the RBC, WBC, hemoglobin, antibodies, lysozyme activity, and phagocytic ratio in common carp that were infected with *Aeromonas hydrophila*. Moreover, in hybrid catfish, the WBC could be enhanced with 300 mg/kg *E. hirta* leaf extract [27]. Garden spurge is a plant that is rich in phenolic compounds, which play a significant role in enhancing immune function and antioxidative activity [54]. The ethanolic extract of *E. hirta* was reported as an immunomodulator in striped catfish (*Pangasianodon hypophthalmus*) by Nhu et al. The extract positively affected the lysozyme activity, immunoglobulin levels, and cumulative mortality against bacterial infection [55]. Significant variations were observed in the serum biochemical indices of the experimental fish, particularly in terms of the total protein, albumin, and globulin levels. These indices exhibited an increasing trend in certain groups that received EHE supplementation compared to those that did not. This observation suggests that the bioactive compounds present in the extract may have stimulated the immune responses of the fish. The increase in the total protein, albumin, and globulin levels indicates a potential enhancement in the fish’s innate immune response. These proteins serve as important components in producing immunoglobulins or antibodies in fish, which play a crucial role in bolstering their immune defenses. This aligns with the findings from the study conducted in [56], which suggested that higher levels of total protein, albumin, and globulins are associated with a stronger innate immune response in fish. Likewise, a study involving freshwater catfish (*Pangasius sutchi*) fingerlings observed that the total serum protein, albumin, globulin, and albumin–globulin ratio levels were elevated in groups that were fed diets supplemented with heart-leaved moonseed (*Tinospora cordifolia*) leaf extract [57]. Notably, the levels of serum glucose were influenced by the addition of EHE, particularly in the groups that received 25% to 75% fish meal replacement. These groups exhibited higher levels of serum glucose compared to the groups with 0% and 100% fish meal replacement. This phenomenon may be attributed to the mixed diet composition involving both commercial fish meal and *P. pardalis* meal, which resulted in the heightened activity of digestive enzymes within the gastrointestinal tract. Consequently, the system required increased energy resources. Glucose is recognized as a pivotal product in the cellular respiration process, and glucose molecules play a crucial role in the bioenergetics of animals. These molecules are subsequently converted into adenosine triphosphate through cellular respiration, thereby serving as a primary energy source. Higher levels of glucose are typically maintained in frogs due to the breakdown of glycogen in the liver, which is followed by the conversion of glucose molecules into energy through glycogenolysis as part of the cellular respiration process [58]. Meanwhile, the assessment of liver function indicated the levels of ALT and AST in all treatment groups did not differ significantly. These results suggest that the EHE used in this study had a discernible impact on the liver function of the fish. The elevated levels of ALT and AST in the blood circulation may indicate that the fish were experiencing some form of stress, resulting in liver stress and potential damage, suggesting that elevations of ALT and AST are key indicators of liver impairment and stress in fish [59]. Moreover, AST is considered one of the three most important myocardial enzymes. It is often utilized as an indicator of cardiac function. The findings of this study suggest that the hearts of fish may not be adversely affected or experience injury when exposed to *E. hirta* extract [60]. These results are in line with a study by Shi et al. concerning yellow catfish (*Pelteobagrus fulvidraco*) that were supplemented with brown macroalgae (*Sargassum horneri*) at levels ranging from 0.1% to 0.4% [61]. The plant extract exhibited improvements in growth performance. Notably, this supplementation led to a significant increase in the levels of serum total protein and a significant decrease in the content of serum total cholesterol and triglyceride. It is also worth mentioning that the levels of ALT and AST remained unchanged when compared to the control group.

## 5. Conclusions

This study represents the first investigation focused on utilizing the alien fish species *P. pardalis* for fish meal production and its subsequent replacement of commercial fish meal in the diets of Chinese bullfrogs (*H. chinensis*). The results obtained from the assessment of the growth performance, organosomatic indices, some hematological parameters, and serum biochemical indices suggest that CFM could be replaced effectively by PPM at levels of up to 75% in the diet, without detrimental effects on the growth performance or organosomatic indices. Furthermore, the inclusion of 300 mg/kg of EHE in the diets led to reduce IF, increased WBCs, and some improved serum biochemical indices, especially the total protein, albumin, and globulin levels. Importantly, the liver function enzymes ALT and AST remained unaffected by this supplementation. Further research could explore the optimum dose of EHE for improved growth performance and other relevant indices in Chinese bullfrogs. Furthermore, the results of this experiment could serve as a guideline for the effective transformation of invasive alien fish species, which pose significant environmental threats, into a valuable resource for the aquaculture industry.

## Figures and Tables

**Table 1:** Proximate composition (in % dry matter) of *Pterygoplichthys pardalis* meal and commercial fish meal.

Proximate Composition	*P. pardalis* Meal (% in Dry Weight)	Commercial Fish Meal (% in Dry Weight)
Moisture	4.43 ± 0.52	4.99 ± 1.13
Ash	16.64 ± 0.43	28.43 ± 0.01
Protein	62.13 ± 0.25	59.07 ± 1.00
Lipid	6.40 ± 0.53	7.40 ± 0.05
Fiber	0.507 ± 0.002	0.507 ± 0.003
Nitrogen-free extract	9.89 ± 0.58	9.6 ± 0.73

Note: Values are expressed as mean ± SD (*n* = 3).

**Table 2:** Ingredients and proximate compositions of the 6 experimental diets.

Ingredient Composition (%)	Experimental Diet (Dry Weight)					
	0	0+	25+	50+	75+	100+
Commercial fish meal	25.02	25.02	18.765	12.51	6.255	0
*P. pardalis* meal	0	0	6.255	12.51	18.765	25.02
Soybean meal	25.03	25.03	25.03	25.03	25.03	25.03
Corn starch	16.55	16.55	16.55	16.55	16.55	16.55
Rice bran	16.55	16.55	16.55	16.55	16.55	16.55
Broken rice	16.55	16.55	16.55	16.55	16.55	16.55
Premix	0.3	0.3	0.3	0.3	0.3	0.3
300 mg/kg *Euphorbia hirta* extract	−	+	+	+	+	+
Total (%)	100	100	100	100	100	100
Proximate Composition (% in Dry Weight)						
Moisture	3.67 ± 2.07	2.28 ± 0.17	1.79 ± 0.01	2.39 ± 0.24	2.13 ± 0.03	2.48 ± 0.22
Ash	10.52 ± 2.78	12.32 ± 2.38	14.63 ± 0.06	10.36 ± 0.16	13.92 ± 0.58	12.46 ± 2.16
Protein	31.38 ± 0.66	31.13 ± 0.89	32.76 ± 1.96	32.83 ± 3.09	33.82 ± 1.69	34.07 ± 1.67
Lipid	11.67 ± 1.04	14.25 ± 0.35	13.33 ± 2.25	11.75 ± 1.06	11.57 ± 0.38	11.23 ± 0.31
Fiber	2.48 ± 0.22	5.16 ± 1.00	5.20 ± 0.37	5.33 ± 0.34	5.44 ± 0.26	5.62 ± 0.13
Nitrogen-free extract	40.28 ±0.23	34.85 ± 2.14	32.28 ± 1.32	37.34 ± 0.25	33.12 ± 1.14	34.13 ±1.56

Note: Premix 1 kg contained vitamins, including Vitamin A 36,000 IU, Vitamin D3 9000 IU, Vitamin E 187 mg, Vitamin K 19 mg, Vitamin B1 52 mg, Vitamin B2 97 mg, Vitamin B6 46 mg, Vitamin C (coated) 69,800 mg, Vitamin B12 60 mcg, pantothenic acid 93 mg, niacin 130 mg, folic acid 10 mg, inositol 225 mg, and biotin 450 mcg, as well as minerals, including Mg 105 mg, Cu 9 mg, Fe 90 mg, Zn 90 mg, I 1.8 mg, Co 450 mcg, Mg 1900 mg, Se 150 mcg, Na 117 mg, K 3600 mg, and Ca 219 mg. Values are expressed as mean ± SD (*n* = 3). (−); the extract was not added, (+); the extract was added.

**Table 3:** Phytochemical composition of *Euphorbia hirta* extract.

Phytochemical	Result	Phytochemical	Result
Alkaloids	−	Phenolics	+
Steroids	−	Flavonoids	−
Triterpenoids	+	Anthraquinones	−
Saponins	−	Cyanogenic glycosides	−
Cardiac glycosides	−	Carbohydrates	+
Coumarins	+		

Note: (+) present, (−) absent.

**Table 4:** Total phenolic content, total flavonoid content, and antioxidant capacity of *Euphorbia hirta* extract.

Analysis	Total Phenolics (mg GAE/g Extract)	Total Flavonoids (mg QE/g Extract)	Antioxidant Capacity (IC_50_) (μg/mL)
*E. hirta* extract	573.58 ± 29.83	39.17 ± 2.90	11.06 ± 6.54
Ascorbic acid	-	-	4.73 ± 1.85

Values are presented as mean ± SD (*n* = 3). IC_50_ = the concentration of the test sample that produces 50% inhibition.

**Table 5:** Amino acid profiles of *Pterygoplichthys pardalis* meal and commercial fish meal.

Amino Acid Composition	Ingredient (mg/100 g)	
	*P. pardalis* Meal	Commercial Fish Meal
**Essential amino acids (EAA**)		
Arginine	6184.61	3859.79
Histidine	2430.52	1359.29
Isoleucine	4444.77	2400.27
Leucine	7943.66	4196.22
Lysine	9114.96	4316.96
Phenylalanine	4210.00	2491.27
Threonine	4585.82	2562.71
Tryptophan	777.76	402.73
Valine	5269.75	3044.43
Methionine	2953.96	1623.08
**ΣEAA**	**47,915.81**	**26,256.75**
**Non-essential amino acids (NEAA)**		
Alanine	5716.19	4209.99
Aspartic acid	10,089.22	5522.03
Glutamic acid	16,280.00	9027.22
Glycine	4955.22	4944.43
Hydroxyproline	604.04	1204.32
Proline	3693.46	2961.60
Serine	4117.72	2399.07
Tyrosine	3496.56	1870.37
Cystine	978.82	541.9
**ΣNEAA**	**49,931.23**	**32,680.93**

**Table 6:** Fatty acid profiles of *Pterygoplichthys pardalis* meal and commercial fish meal.

Fatty Acid Composition	Ingredient (g/100 g)	
	*P. pardalis* Meal	Commercial Fish Meal
**Saturated fatty acid (SFA)**		
Lauric acid (C12:0)	0.02	0.02
Myristic acid (C14:0)	0.07	0.41
Pentadecanoic acid (C15:0)	0.02	0.09
Palmitic acid (C16:0)	0.41	1.95
Heptadecanoic acid (C17:0)	0.01	0.12
Stearic acid (C18:0)	0.18	0.75
Arachidic acid (C20:0)	Not detected	0.06
Heneicosanoic acid (C21:0)	Not detected	0.01
Behenic acid (C22:0)	0.01	0.04
Lignoceric acid (C24:0)	Not detected	0.04
**ΣSFA**	**0.72**	**3.49**
**Unsaturated fatty acid (UFA)**		
Trans-fatty acid	Not detected	0.17
Omega 3	0.06	0.84
Omega 6	0.21	0.36
Omega 9	0.35	0.83
**ΣUFA**	**0.62**	**2.20**
**Monounsaturated fatty acid (MUFA)**		
Palmitoleic acid (C16:1n-7)	0.01	0.53
Cis-9-oleic acid (C18:1n-9c)	0.35	0.01
Cis-10-heptadecenoic acid (C17:1n10)	Not detected	0.04
Trans-9-elaidic acid (C18:1n9t)	Not detected	0.02
Cis-9-oleic acid (C18:1n9c)	Not detected	0.77
Cis-11-eicosenoic acid (C20:1n11)	Not detected	0.05
Erucic acid (C12:1n9)	Not detected	0.01
Nervonic acid (C24:1n9)	Not detected	0.05
**ΣMUFA**	**0.36**	**1.48**
**Polyunsaturated fatty acid (PUFA)**		
Trans-linolelaidic acid (C18:2n6t)	Not detected	0.15
Cis-9-12-linoleic acid (C18:2n6)	0.11	0.12
Gamma-linolenic acid (C18:3n6)	0.02	0.01
Alpha-linoleic acid (C18:3n3)	0.01	0.07
Cis-11,14-eicosatrienoic acid (C20:2n6)	Not detected	0.03
Cis-8,11,14-eicosatrienoic acid (C20:3n6)	0.03	0.04
Arachidonic acid (C20:4n6)	0.06	0.19
Cis-5,8,11,14,17-eicosapentaenoic acid (C20:5n3)	0.01	0.28
4,7,10,13,16,19-Docosahexaenoic acid (C22:6n3)	0.04	0.49
**ΣPUFA**	**0.28**	**1.38**

## Data Availability

Data are contained within the article.
